# SML resist processing for high-aspect-ratio and high-sensitivity electron beam lithography

**DOI:** 10.1186/1556-276X-8-139

**Published:** 2013-03-27

**Authors:** Mohammad Ali Mohammad, Steven K Dew, Maria Stepanova

**Affiliations:** 1 Department of Electrical and Computer Engineering, University of Alberta, Edmonton, Alberta, T6G 2V4, Canada; 2 National Institute for Nanotechnology NRC, 11421 Saskatchewan Drive, Edmonton, Alberta, T6G 2M9, Canada

**Keywords:** SML resist, Electron beam lithography, High-aspect-ratio nanolithography, Nanolithography, Nanofabrication, Lift-off, Preventing pattern collapse, Resists (85.40.Hp), Electron beam lithography (85.40.Hp), Nanolithography (81.16.Nd)

## Abstract

A detailed process characterization of SML electron beam resist for high-aspect-ratio nanopatterning at high sensitivity is presented. SML contrast curves were generated for methyl isobutyl ketone (MIBK), MIBK/isopropyl alcohol (IPA) (1:3), IPA/water (7:3), *n*-amyl acetate, xylene, and xylene/methanol (3:1) developers. Using IPA/water developer, the sensitivity of SML was improved considerably and found to be comparable to benchmark polymethylmethacrylate (PMMA) resist without affecting the aspect ratio performance. Employing 30-keV exposures and ultrasonic IPA/water development, an aspect ratio of 9:1 in 50-nm half-pitch dense grating patterns was achieved representing a greater than two times improvement over PMMA. Through demonstration of 25-nm lift-off features, the pattern transfer performance of SML is also addressed.

## Background

Fabrication of nanoscale structures and devices such as nanoimprint lithography templates, dynamic random-access memory capacitors, zone plates (X-ray lenses), etc. requires a high-aspect-ratio (AR) and high-resolution patterning capability. Utilizing electron beam lithography (EBL) to fabricate such nanostructures further requires that the patterning be performed as rapidly as possible (high throughput) due to the serial writing nature of EBL. The requirement of high throughput often imposes a trade-off between the selection of processing conditions and performance. As an example, using a higher voltage in EBL enables the fabrication of higher AR nanostructures; however, the electron dose increases in proportion to the voltage, thus increasing the time of exposure. Careful selection of other processing parameters such as using a higher performance developer solution can decrease the electron dose requirement (increase the process sensitivity) and, to a certain extent, compensate for such trade-offs.

The well-known positive-tone resists polymethylmethacrylate (PMMA) and ZEP-520 (Zeon Corporation, Tokyo, Japan) can be patterned with sub-20-nm resolution for dense grating patterns. However, the achievable ARs of PMMA on solid substrates are limited to 2:1 to 4:1 at 25 keV [1,2], to approximately 5:1 at 50 keV [1,3], and to 12:1 to 20:1 at 100 keV [1,4,5]. Similarly, ZEP resist has ARs limited to 4:1 at 20 keV [6] and to 7:1 at 100 keV [7], albeit with over three times higher sensitivity than PMMA. Another positive-tone resist, polymethylglutarimide (PMGI), has been patterned with an AR of over 2:1 at 30 keV [8] and extremely high AR of 38:1 at 100 keV [9] using an optimized development process. However, the sensitivity of PMGI is four to nine times lower than that of PMMA, requiring up to 18,000 μC/cm^2^[9] to expose a single line. Similar trends are observed for negative-tone resists such as hydrogen silsesquioxane (HSQ). Reported ARs for HSQ are 4:1 at 10 keV [10], 7:1 at 50 keV [11], and 25:1 at 100 keV [12,13]. HSQ’s main attraction is its extremely high resolution (<10 nm); however, its sensitivity is usually an order lower than that of PMMA. Other negative-tone resists such as AZ nLOF 2020 (Clariant Corporation, Muttenz, Switzerland) [14] and high molecular weight polystyrene (PS) [15] have sensitivities a fraction of that of PMMA; however, their AR performance is limited to 4:1 to 5:1 at 100 keV for AZ nLOF 2020 [14] and to less than 2:1 at 20 keV for PS [15,16].

Recently, an EBL resist ‘SML’ [17] has been introduced by EM Resist Ltd. (Macclesfield, UK) in thicknesses ranging from 50 to 2,000 nm. SML is a positive-tone, organic resist that has been designed for high-AR patterning. The resist is anticipated to yield ARs of up to 10:1 at 30 keV and exceeding 50:1 at 100 keV [17]. This represents a greater than two times improvement over benchmark PMMA resist; however, its sensitivity and resolution are lower than those of PMMA using supplier-recommended conditions. Similar to other positive-tone resists such as PMMA [18], PMGI [8], and ZEP [19], SML may be developed in methyl isobutyl ketone (MIBK)/isopropyl alcohol (IPA) (1:3) solution and rinsed in IPA [20].

In this work, a systematic experimental study of SML as a high-performance EBL resist at 30 keV is conducted with the aim of co-optimizing sensitivity, contrast, and AR. A total of six developers (both single- and binary-component) are evaluated by generating the contrast curves and comparing their respective sensitivities and contrast values. After selecting the developer with desired characteristics, high-AR grating patterns at various pitch values are fabricated to obtain a dense, high-AR, and high-sensitivity nanolithography process. The pattern transfer performance of SML is also explored by lift-off experiments. At each stage of this work, the performance of SML resist is compared to that of PMMA.

## Methods

The SML samples used in this study were provided courtesy of EM Resist Ltd. [17] as pre-spun and baked chips. The experimental work with SML resist began using supplier-recommended conditions [17,20] to fabricate grating structures in 300- and >1,500-nm-thick resist samples. Based on the understanding of the resist gained in these experiments, the majority of the work was conducted in three sequential steps: (a) generation of SML contrast curves with six different developers, followed by (b) fabrication and characterization of high-AR gratings using a selected developer, and (c) evaluation of lift-off performance.

To generate the contrast curves, an array of 20 × 75 μm rectangular pads (spaced by 20 μm) with a gradually increasing dose was exposed to 30-keV electrons (Raith 150^TWO^, Dortmund, Germany) on 300- to 330-nm-thick SML resist samples. The exposed samples were developed for 20 s at ambient temperature in six developers: MIBK, MIBK/IPA (1:3), IPA/water (7:3), *n*-amyl acetate, xylene, and xylene/methanol (3:1). The developed samples were quickly dried in a nitrogen flow, and no post-development rinsing was performed. The resulting resist surfaces were scanned using a physical profilometer (KLA-Tencor Alpha-Step IQ, Milpitas, CA, USA) having a depth resolution of 10 nm.

To fabricate dense, high-AR gratings, large arrays of 50- to 200-nm-pitch grating patterns were exposed at 30 keV on 300- to 330-nm-thick SML samples. An exposure voltage of 30 keV (the highest voltage on Raith 150^TWO^ EBL system) was selected to maximize the AR while achieving high sensitivity through the development process. The width of the grating arrays were kept sufficient for capturing the contribution of proximity effects. The exposure current was 23 to 24 pA (7.5-μm aperture), and a step size of 2 nm was used. The exposed samples were developed ultrasonically for 20 s in IPA/water (7:3) (developer selected after contrast curve study). Before drying the samples in flowing nitrogen, the developed samples were briefly (approximately 2 s) immersed in a low-surface-tension fluid (pentane or hexane) to reduce the probability of pattern collapse. Prior to scanning electron microscope (SEM) imaging, the samples were coated with a 6-nm chromium layer (Gatan PECS, Pleasanton, CA, USA). Cleaved samples were coated at a 45° tilt with the sample cross section facing the target. The SEM imaging (Hitachi S-4800, Schaumburg, IL, USA) was conducted at 5 keV, 20 μA, and 4-mm working distance. To evaluate the pattern transfer capability of SML resist, metal lift-off was performed. By electron beam evaporation, 50 nm of chromium was deposited on nanoscale SML gratings and the resulting stack lifted-off by immersing for 1 min in an ultrasonic acetone bath.

## Results and discussion

**Figure 1:**
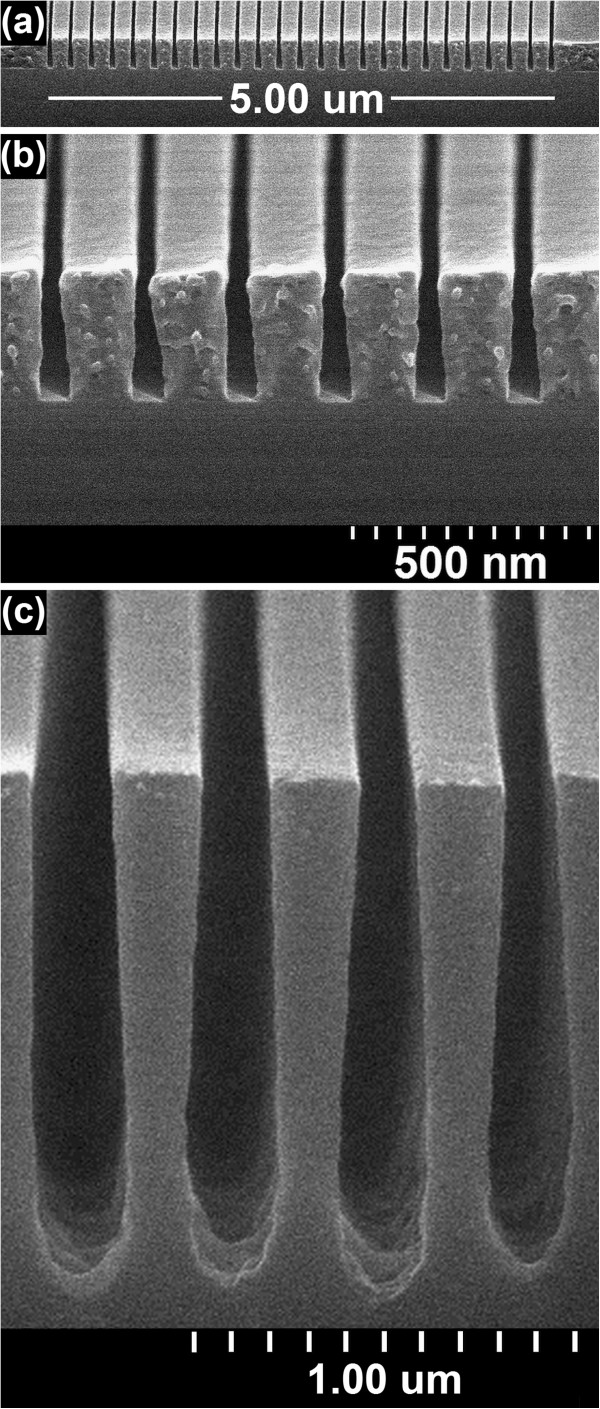
**Cross-sectional micrographs of SML exposed at 30 keV and developed in MIBK/IPA (1:3) for 20 s.** The panels show (**a**) 5-μm array of 200-nm-pitch gratings in 300-nm-thick resist, (**b**) magnified image with thickness of 282 nm and line widths of 45 to 67 nm from top to bottom of gratings, and (**c**) 400-nm-pitch gratings in >1,500-nm-thick resist (no clearance) with the achieved depth of 1,380 nm and line widths of 180 to 220 nm from top to bottom of gratings. The exposure doses were (**a**, **b**) 3.6 nC/cm and (**c**) 700 μC/cm^2^, and the aspect ratios ranged from (**a**, **b**) 4.2 to 6.3 and (**c**) 6.3 to 7.7. The resist was cleaved and coated with a 6-nm Cr layer before imaging.

Figure 1 presents cross-sectional micrographs of cleaved gratings fabricated in SML using the supplier-recommended developer, MIBK/IPA (1:3). SML was found to be easy to use, and it was possible to readily fabricate gratings with an AR better than PMMA in introductory attempts with both 300- (Figure 1a,b) and >1,500-nm-thick (Figure 1c) films. In Figure 1a, a uniform 5-μm-wide array of 200-nm-pitch gratings is patterned at an exposure line dose of 3.6 nC/cm. In comparison, similar PMMA gratings can be fabricated using approximately three times higher sensitivity. Figure 1c shows a magnified image from the center of the array measuring a thickness of 282 nm and line widths ranging from 45 to 67 nm (from top to base of gratings), resulting in ARs of 4.2 to 6.3. In Figure 1c, an array of 400-nm-pitch gratings is patterned to a depth of 1,380 nm (no clearance) using an exposure area dose of 700 μC/cm^2^. From top to bottom, the line widths range from 180 to 220 nm, resulting in ARs of 6.3 to 7.7. The AR results achieved using MIBK/IPA (1:3) are not optimized and can be significantly improved; however, the much lower sensitivity compared to PMMA requires a higher sensitivity developer that maintains or even improves the AR performance.

**Figure 2:**
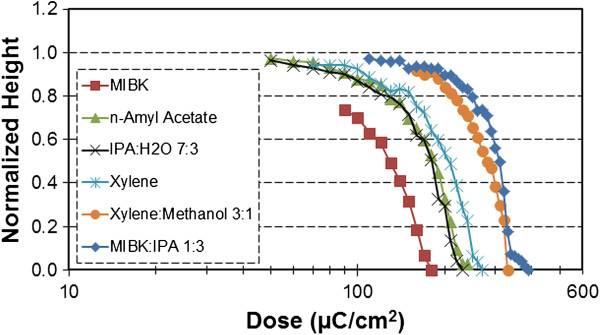
**SML contrast curves generated using 30 keV on 300- to 330-nm-thick resist.** The development was performed for 20 s in MIBK (squares), *n*-amyl acetate (triangles), IPA/water (7:3) (crosses), xylene (stars), xylene/methanol (3:1) (circles), and MIBK/IPA (1:3) (diamonds).

The SML contrast curves for the six developers: MIBK, MIBK/IPA (1:3), IPA/water (7:3), *n*-amyl acetate, xylene, and xylene/methanol (3:1) are presented in Figure 2. The contrast (*γ*) was measured using the standard definition *γ* = [log(*D*_0_ / *D*_1_)]^-1^, where the clearance (*D*_0_) and onset (*D*_1_) doses were determined by extending the tangent of the largest slope to the 0, 1 intercepts of the ordinate axis. Comparing the contrast curves of the supplier-recommended MIBK/IPA (1:3) to MIBK, it was found that using undiluted MIBK yields a 54% higher sensitivity at the cost of a similar (53%) contrast loss. The other four developers exhibit a sensitivity and contrast performance between those of MIBK/IPA (1:3) and MIBK. In particular, two developers, *n*-amyl acetate and IPA/water (7:3), provide a relatively high sensitivity and contrast as compared to the other developers. The surfaces of the developed patterns were also inspected by optical microscopy, and it was found that all of the developers provide a uniform thickness loss with increasing dose except for xylene/methanol (3:1). Using xylene/methanol (3:1), the dissolution is non-uniform with certain exposed areas dissolving more rapidly than others, leaving a porous resist surface. Perhaps a technique such as ultrasonic agitation may be useful in this regard. An additional document [see Additional file 1] compares (a) SML contrast curves at 10 and 30 keV and (b) the clearance dose at 10, 20, and 30 keV, for selected developers.

**Figure 3:**
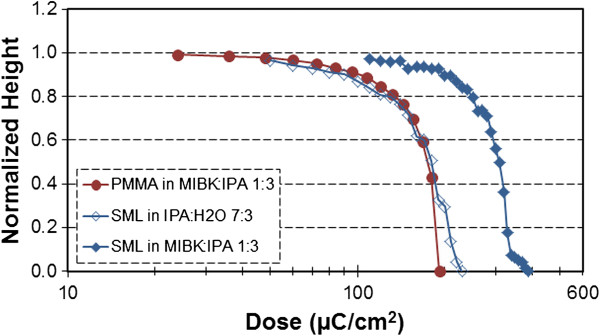
**Comparison of SML and PMMA contrast curves.** Both SML (triangles) and PMMA (circles) were exposed at 30 keV and developed for 20 s in MIBK/IPA (1:3) (filled symbols) and IPA/water (7:3) (open symbols).

**Figure 4:**
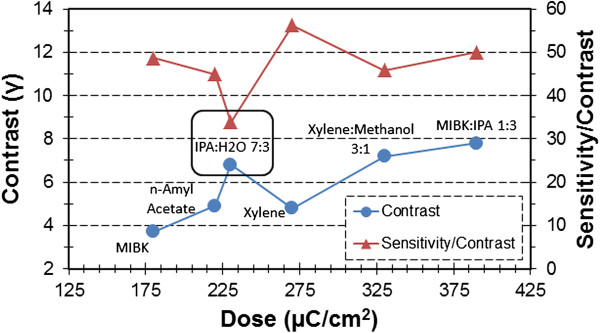
**Comparison of SML contrast and contrast-weighted sensitivity for various developers.** The contrast (circles) and contrast-weighted sensitivity (triangles) have been arranged in increasing clearance dose. The contrast-weighted sensitivity has units of dose (μC/cm^2^).

In Figure 3, comparing the contrast curves of SML and PMMA, both developed in MIBK/IPA (1:3) for 20 s, it was found that SML is 71% less sensitive than PMMA and has a 7% higher contrast. However, when SML is developed in IPA/water (7:3), a 41% sensitivity improvement is realized as compared to SML in MIBK/IPA (1:3), enabling the sensitivity of SML to be comparable to that of PMMA in MIBK/IPA (1:3). This behavior is similar to PMMA - the sensitivity of PMMA developed in IPA/water (7:3) improves by 30% as compared to PMMA developed in MIBK/IPA (1:3) [21]. The sensitivity improvement of SML is achieved with a minor trade-off in contrast - SML in IPA/water (7:3) has a 13% lower contrast than SML in MIBK/IPA (1:3). The IPA/water (7:3) mixture provides the highest contrast versus sensitivity trade-off. By arranging SML developers with increasing clearance dose as shown in Figure 4, it was found that IPA/water (7:3) has a higher-than-average contrast and the best *contrast-weighted sensitivity*. The quantity contrast-weighted sensitivity has been introduced as our figure of merit to factor in sensitivity while selecting the developer with the best contrast. The IPA/water developer has other merits including cost, safety, and experience of the EBL community using it as a developer for PMMA [1,19,21] and ZEP [19,22] at both ambient and cold development conditions. In addition to the aforementioned developers, the development of SML in MIBK/IPA (1:3) at -15°C cold development conditions was also attempted; however, due to the extremely low sensitivity (clearance onset >1,000 μC/cm^2^), it was abandoned. An additional document [see Additional file 2] compares the contrast-weighted sensitivity of SML to the six other resists cited in the ‘Background’ section.

Based on the analysis of contrast curves, IPA/water (7:3) was selected as the preferred developer for fabricating dense, high-AR gratings. Similar to PMMA, both IPA and water alone are poor or non-developers for SML resist but are effective in combination. The usage of ultrasonic agitation during development was chosen to help promote the dissolution of SML fragments as inspired by Yasin’s work [21]. Since resist fragments tend to coil in poor solvents and exhibit a smaller radius of gyration, ultrasonic agitation may be expected to promote the rapid removal of these fragments, enabling a narrower grating trench [21]. As described in the ‘Methods’ section, a brief rinse in low-surface-tension fluid was used to reduce the probability of pattern collapse. The surface tension of pentane (approximately 16 dyn/cm) and hexane (approximately 18 dyn/cm) is at least four times less than that of water (approximately 73 dyn/cm).

**Figure 5:**
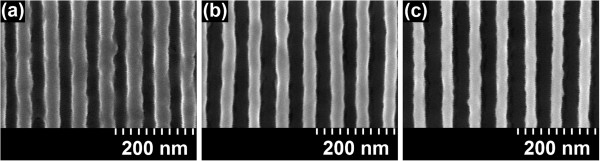
**Micrographs of 70-nm-pitch gratings patterned by 30 keV on 300- to 330-nm-thick SML.** Effect of dose on increasing line width (**a**) 550 pC/cm, 25-nm gap, (**b**) 750 pC/cm, 32-nm gap, and (**c**) 950 pC/cm, 40-nm gap. Data obtained for 20 s ultrasonic development in IPA/water (7:3) and 2 s pentane rinse.

Figure 5 presents top-view grating micrographs of 70-nm-pitch SML gratings in a 300- to 330-nm-thick resist showing the effect of increasing line dose. The line width increases from 25 nm at 550 pC/cm (Figure 5a) to 32 nm at 750 pC/cm (Figure 5b) and to 40 nm at 950 pC/cm (Figure 5c) just prior to pattern collapse. Observing the top-view grating micrographs, clearance cannot be conclusively ascertained; however, this question is explored through cross-sectional micrographs ahead. Based on the observations from Figure 5, it is estimated that as low as 25-nm resolution with SML is readily achievable without resolution enhancement techniques. Furthermore, the gratings show low line edge roughness. The resolution limits (with thinner resists) were not explicitly pursued as this work focused on maximizing the AR, pattern density, and sensitivity by co-optimizing the exposure and development conditions. Given that the proximity effect appears to be of minor importance, if at all (see Figure 1a), the results in Figure 5 are representative of the resist performance even without clearance and can be employed to co-optimize the resist thickness and process conditions if so desired.

**Figure 6:**
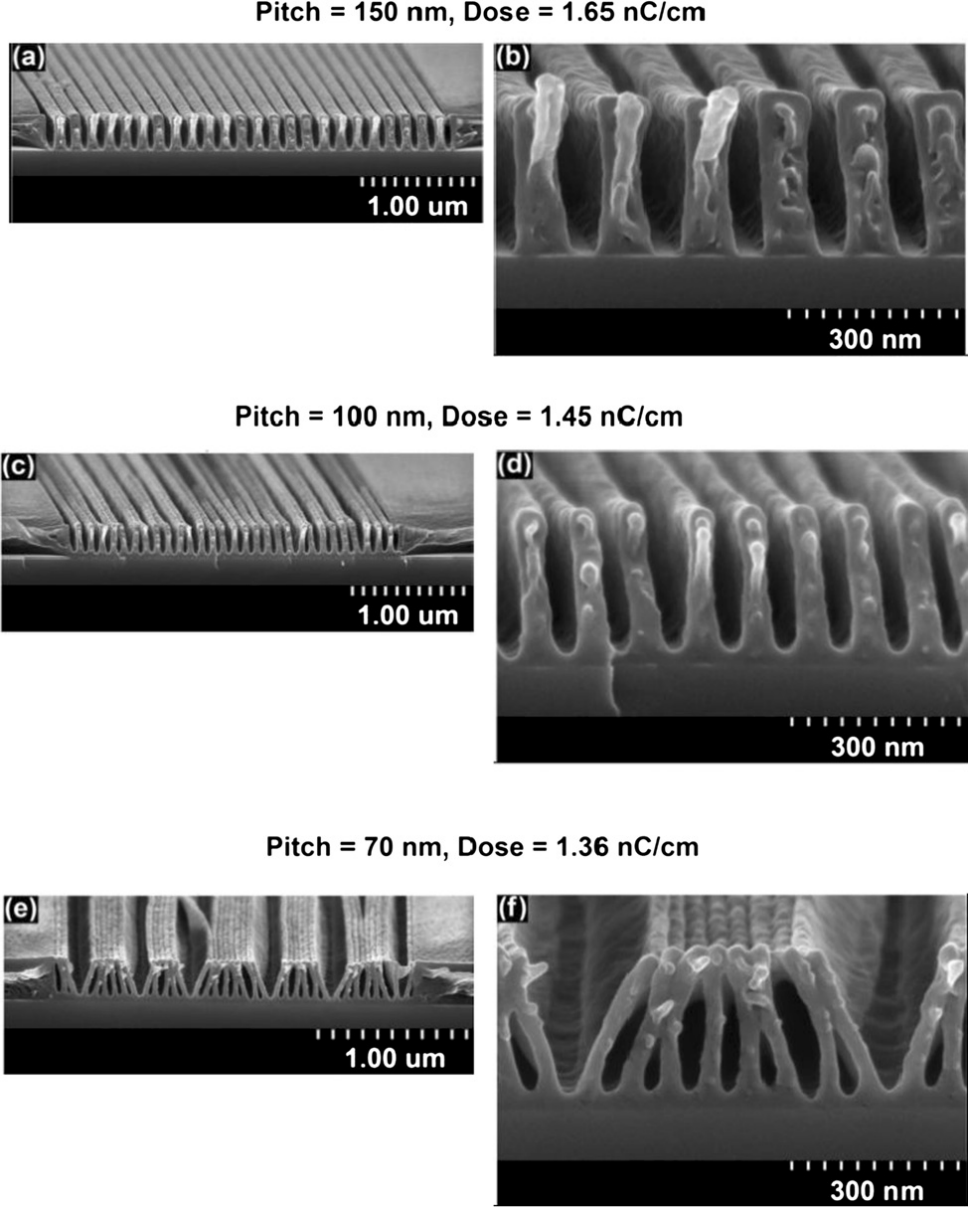
**Cross-sectional micrographs of SML exposed at 30 keV on 300- to 330-nm-thick resist.** Achievable line width and pitch (**a**, **b**) 36- to 40-nm gaps in 150-nm pitch, (**c**, **d**) 33- to 40-nm gaps in 100-nm pitch, and (**e, f**) 30-nm sidewall in 70-nm pitch, yielding an approximate AR of 9:1 in all cases. The development procedure is identical to that in Figure 5. The resist was cleaved and coated with a 6-nm Cr layer before imaging.

In Figure 6, micrographs of cleaved SML resist are presented showing the effect of reducing the grating pitch from 150 (Figure 6a,b) to 100 nm (Figure 6c,d) and finally to 70 nm (Figure 6e,f). All micrographs are captured at a SEM tilt of 14° from normal. The upper row of micrographs (Figure 6a,c,e) shows the complete patterned arrays, and the lower row of micrographs (Figure 6b,d,f) shows zoomed-in micrographs taken near the center of the grating arrays. Observing the complete arrays, the gratings are uniform and no proximity effect can be noticed. This result is significant as resists such as PMMA, at comparable conditions, exhibit wider pattern features and/or collapse in the center of the grating arrays as compared to the sides. It was observed that denser gratings require a higher dose for clearance and the resolution also improves. The highest density gratings that could be fabricated before pattern collapse were of 100-nm pitch in a 300- to 330-nm-thick resist. In addition, 80-nm-pitch gratings were also patterned (not shown); however, those also collapsed. From the micrographs in Figure 6a,b,c,d,e,f, feature sizes between 30 and 40 nm are observed yielding a best case AR of 9:1 at 30 keV for all pitch values. It is clear that for 30-keV exposures, this AR is two to five times better than the resists reviewed in the ‘Background’ section.

The SEM imaging with SML is quite challenging. Dense grating structures deform and bend as a result of the scanning accompanied by visible film shrinkage. The gratings shown in Figure 6a,b,c,d had perfectly vertical sidewalls before a 5-s SEM scan. The film shrinkage also reduces the AR measurement. Thick (>1,500 nm) patterned SML films show exaggerated deformation and, in some cases, tearing and de-lamination. An additional document explains the visualization challenge and mitigation strategies in more detail [see Additional file 3]. We would like to re-iterate that the resist deformation is a SEM visualization issue, and not the result of EBL exposure.

**Figure 7:**
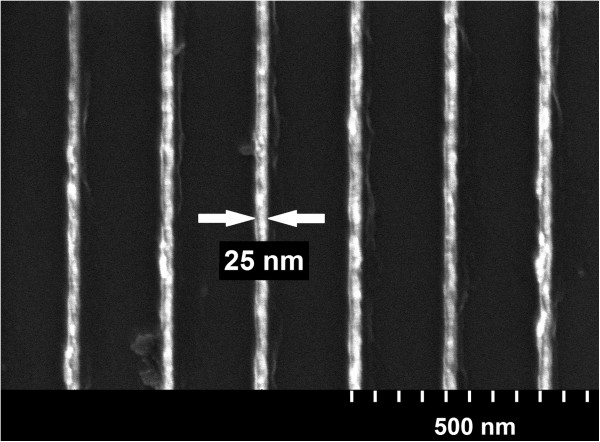
**Micrograph of 25-nm-wide lifted-off Cr gratings.** The metallization (50-nm thickness) was performed by e-beam evaporation.

Finally, the lift-off procedure using SML was found to be very efficient. Un-patterned SML may be readily stripped by acetone when rinsed with a wash bottle for a few seconds. Patterned SML with 50 nm of chromium metal was fully removed by acetone by immersing in an ultrasonic bath for 1 min. Figure 7 shows 25-nm-wide chromium lines in a 200-nm-pitch grating pattern exposed at 1,650 pC/cm. Considering that the chromium was deposited on a 300- to 330-nm-thick resist film, this result implies that an even higher AR (≥12:1) may have been obtained previously than observed (≥9:1) during cross-sectional SEM due to the fragility of the resist.

## Conclusions and recommendations

A detailed characterization of SML electron beam resist has been presented with focus on high-aspect-ratio nanopatterning at high sensitivity. Contrast curves of six developers: MIBK, MIBK/IPA (1:3), IPA/water (7:3), *n*-amyl acetate, xylene, and xylene/methanol (3:1), were compared for the highest contrast and sensitivity. SML’s pattern density limits and lift-off capability were also evaluated.

SML was found to be a capable and versatile EBL resist. Aspect ratios of at least 9:1 are possible at 30 keV, suggesting over 100% improvement as compared to PMMA or ZEP. IPA/water (7:3) was found to be the most suitable developer for high-contrast and high-sensitivity nanopatterning. Using IPA/water (7:3) developer, SML’s sensitivity is close to PMMA and therefore represents a 40% improvement in sensitivity over existing SML results. Metal lift-off was found to be easy and efficient.

Based on the experiences gained through this research, the following recommendations are offered for further work with SML: (a) to find a stronger developer (stronger than MIBK) and combine it with a small molecule non-solvent such as methanol, (b) to develop pattern collapse prevention techniques such as supercritical drying [23] with exchange liquid other than IPA and/or use of surfactants [24], and (c) to invest efforts to find damage-free electron microscopy imaging conditions.

## Abbreviations

AR: aspect ratio; EBL: electron beam lithography; HSQ: hydrogen silsesquioxane; IPA: isopropyl alcohol; MIBK: methyl isobutyl ketone; PMGI: polymethylglutarimide; PMMA: polymethylmethacrylate; PS: polystyrene; SEM: scanning electron microscope

## Competing interests

The authors declare that they have no competing interests.

## Authors’ contributions

MAM designed and performed the fabrication and characterization experiments, analyzed the data, and drafted the manuscript. SKD analyzed the contrast and sensitivity data and critically revised the manuscript. MS conceived the study and helped in the drafting and revision of the manuscript. All authors read and approved the final manuscript.

## Supplementary Material

Additional file 1: Figure A1 SML (a) contrast curves, and (b) clearance dose trends for various voltages and developers. The developers used are MIBK:IPA 1:3 (filled symbols) and IPA:Water 7:3 (open symbols), for 20 sec each, showing (a) contrast curves at 10 keV (triangles) and 30 keV (circles), and (b) clearance dose vs. voltage (squares). The data has been acquired through optical profilometry (Zygo NewView 5000). Click here for file

Additional file 2: Table T1 Comparison of contrast weighted sensitivity of various resists. Click here for file

Additional file 3: Figures A2 and A3 Figure A2. Adverse effects of SEM imaging on SML resist. The panels show (a) swelling and tearing of resist upon low magnification scan, and (b) bending of grating patterns after high magnification scan from center of the same grating patterns. Figure A3. Shrinking of SML resist surface due to SEM imaging. The panels show the micrographs (a) after first scan at low magnification, and (b) after second scan at high magnification. Observe the unexposed surfaces alongside the grating patterns. Click here for file
