# Supplementary material for: SML resist processing for high-aspect-ratio and high-sensitivity electron beam lithography
Source: Nanoscale Res Lett. 2013 Mar 27;8(1):139. doi: 10.1186/1556-276X-8-139 (PMC3617037; doi:10.1186/1556-276X-8-139)
Supplement: Additional file 1: Figure A1 — SML (a) contrast curves, and (b) clearance dose trends for various voltages and developers. The developers used are MIBK:IPA 1:3 (filled symbols) and IPA:Water 7:3 (open symbols), for 20 sec each, showing (a) contrast curves at 10 keV (triangles) and 30 keV (circles), and (b) clearance dose vs. voltage (squares). The data has been acquired through optical profilometry (Zygo NewView 5000). [file 1556-276X-8-139-S1.pdf]

Additional File 1

SML resist processing for high aspect ratio and high sensitivity electron beam lithography

Mohammad Ali Mohammad, Steven K. Dew, and Maria Stepanova

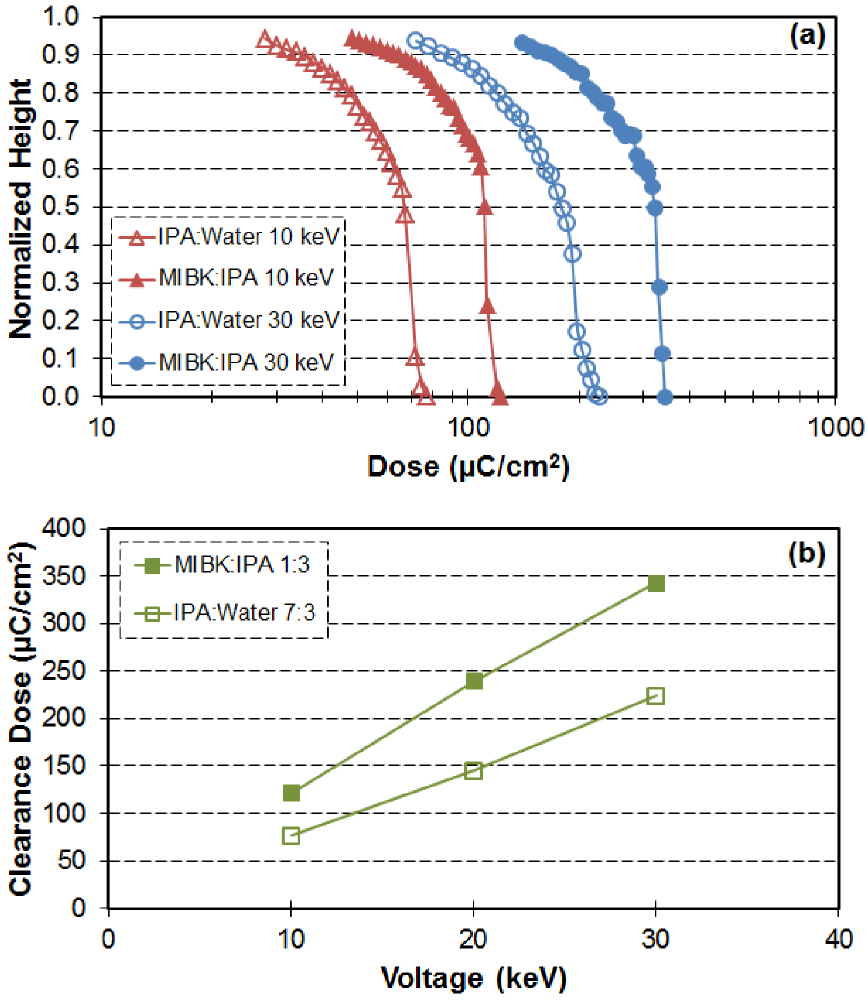

Figure A1. SML (a) contrast curves, and (b) clearance dose trends for various voltages and developers. The developers used are MIBK:IPA 1:3 (filled symbols) and IPA:Water 7:3 (open symbols), for 20 sec each, showing (a) contrast curves at 10 keV (triangles) and 30 keV (circles), and (b) clearance dose vs. voltage (squares). The data has been acquired through optical profilometry (Zygo NewView 5000).
