# Supplementary material for: SML resist processing for high-aspect-ratio and high-sensitivity electron beam lithography
Source: Nanoscale Res Lett. 2013 Mar 27;8(1):139. doi: 10.1186/1556-276X-8-139 (PMC3617037; doi:10.1186/1556-276X-8-139)
Supplement: Additional file 3: Figures A2 and A3 — Figure A2. Adverse effects of SEM imaging on SML resist. The panels show (a) swelling and tearing of resist upon low magnification scan, and (b) bending of grating patterns after high magnification scan from center of the same grating patterns. Figure A3. Shrinking of SML resist surface due to SEM imaging. The panels show the micrographs (a) after first scan at low magnification, and (b) after second scan at high magnification. Observe the unexposed surfaces alongside the grating patterns. [file 1556-276X-8-139-S3.pdf]

Additional File 3

SML resist processing for high aspect ratio and high sensitivity electron beam lithography

Mohammad Ali Mohammad, Steven K. Dew, and Maria Stepanova

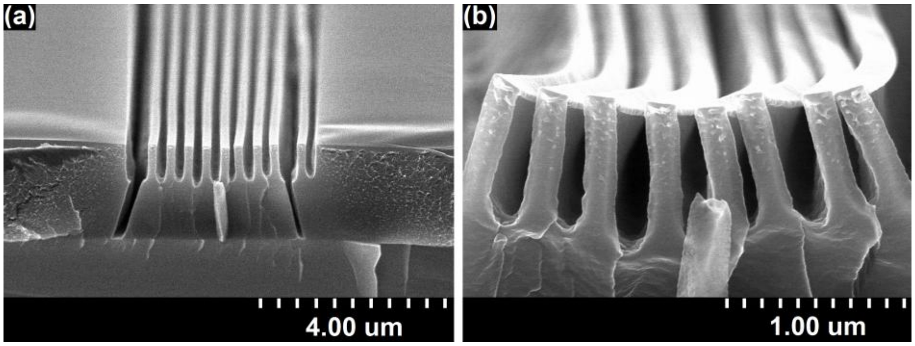

Figure A2. Adverse effects of SEM imaging on SML resist. The panels show (a) swelling and tearing of resist upon low magnification scan, and (b) bending of grating patterns after high magnification scan from centre of the same grating patterns.

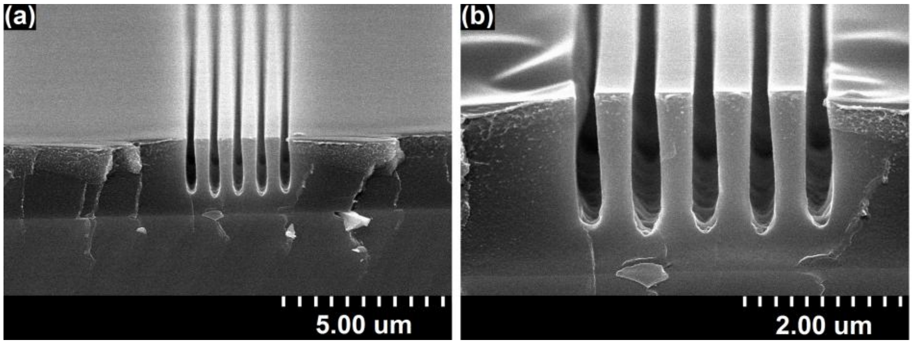

Figure A3. Shrinking of SML resist surface due to SEM imaging. The panels show the micrographs (a) after first scan at low magnification, and (b) after second scan at high magnification. Observe the unexposed surfaces alongside the grating patterns.

The micrographs above have been acquired at 5 keV, 20 µA, 4 mm working distance, and slow scan (20-60 sec). Lowering the imaging voltage would cause less damage; however, the working distance would need to be reduced and image quality would suffer. Increasing the imaging voltage (30 keV) and increasing the working distance (greater depth-of-focus) has also surprisingly enabled less resist damage at the cost of reducing surface information. Faster scans after focusing on nearby objects also helpful.
